# Neuroticism Modifies Psychophysiological Responses to Fearful Films

**DOI:** 10.1371/journal.pone.0032413

**Published:** 2012-03-30

**Authors:** Emmanuelle Reynaud, Myriam El Khoury-Malhame, Jérôme Rossier, Olivier Blin, Stéphanie Khalfa

**Affiliations:** 1 National Center for Scientific Research (CNRS), Timone Neuroscience Institute (INT, UMR 7289), Marseille, France; 2 Department of Neurosciences, Faculty of Life and Health Sciences, University of the Mediterranean Aix Marseille 2, Marseille, France; 3 Institute of Psychology, University of Lausanne, Lausanne, Switzerland; 4 Public Assistance for Marseille Hospitals (APHM), Unit for Clinical Pharmacology and Therapeutic Evaluation (CIC-UPCET), Timone Hospital, Marseille, France; University of Granada, Spain

## Abstract

**Background:**

Neuroticism is a personality component frequently found in anxious and depressive psychiatric disorders. The influence of neuroticism on negative emotions could be due to its action on stimuli related to fear and sadness, but this remains debated. Our goal was thus to better understand the impact of neuroticism through verbal and physiological assessment in response to stimuli inducing fear and sadness as compared to another negative emotion (disgust).

**Methods:**

Fifteen low neurotic and 18 high neurotic subjects were assessed on an emotional attending task by using film excerpts inducing fear, disgust, and sadness. We recorded skin conductance response (SCR) and corrugator muscle activity (frowning) as indices of emotional expression.

**Results:**

SCR was larger in high neurotic subjects than in low neurotics for fear relative to sadness and disgust. Moreover, corrugator activity and SCR were larger in high than in low neurotic subjects when fear was induced.

**Conclusion:**

After decades of evidence that individuals higher in neuroticism experience more intense emotional reactions to even minor stressors, our results indicate that they show greater SCR and expressive reactivity specifically to stimuli evoking fear rather than to those inducing sadness or disgust. Fear processing seems mainly under the influence of neuroticism. This modulation of autonomic activity by neurotics in response to threat/fear may explain their increased vulnerability to anxious psychopathologies such as PTSD (post traumatic stress disorder).

## Introduction

When a person feels an emotion, bodily changes are observed in the activity of the autonomic nervous system (ANS) and brain structures as well as facial expressions [1]. Emotional experience and expression are considered two of the major emotional manifestations [2]. However, the list of components needed to define a particular emotion has been broadened to include interindividual factors of variability such as personality traits. In fact, these traits seem to influence and condition the way we experience and respond to emotions [3]. Most accepted models of personality include the dimension of neuroticism [4]. The characteristics of this measurable trait include a tendency to be worried and anxious [5] and are related to the experience of negative affect [6]–[9]. In fact, subjects with high neuroticism scores are more distressed by negative mood induction than those with low scores and they tend to be more psychologically reactive to stressors [6], [10] Because psychological distress is physiologically translated, recent studies have tried to characterize the psychophysiological correlates of neuroticism.

Initial studies used electromyographic (EMG) measures in different stressful and fearful paradigms [11], [12]. Under fearful conditions, high neurotic subjects showed greater startle reactions than low neurotic subjects. Neuroticism thus appears to potentiate EMG magnitude in a context of fear.

Autonomic reactions are important components of the emotional response. Among them, skin conductance response (SCR) stands out as a classical and sensitive measure reflecting the sympathetic system reactivity. Subjects with high harm-avoidance scores (tendency to inhibit responses to signals of aversive stimuli) who tend to be high in neuroticism showed larger SCR than those with low scores [13]. Moreover, SCR is higher among neurotics than emotionally stable individuals, as neurotic individuals exhibit both greater reactivity and more sustained responses to emotionally unpleasant stimuli than do nonneurotics [14].

In sum, behavioral and physiological studies have shown that neurotics tend to be more psychologically and physiologically reactive to emotionally negative events than controls. This observation is in accordance with the characteristics of neuroticism, since a high level of neuroticism is associated with self-reported negative affective states [15]. More precisely, it has been postulated that the more neuroticism predominates as a central aspect of personality, the more threat will be perceived in a variety of situations [16]. In this perspective, subjects high in neuroticism are vulnerable to stress when conditions are construed as threatening, using an active coping task. The influence of neuroticism on negative emotions was mediated by threat appraisals [17]. Neuroticism was shown to be associated with processing of fear-inducing emotion, but other findings showed that neuroticism is also associated with processing of sad stimuli, suggesting that this trait is highly related to depressed mood. In fact, a high neuroticism score is a predictor of the vulnerability to develop depression and is related to a lifetime prevalence of depression [18]–[21].

Althought neuroticism is a personality component, frequently found in many anxious and depressive psychiatric disorders, the physiological bases of this trait remain largely unknown. To better understand the behavioral and physiological modifications induced by neuroticism on fear and sadness processing as compared to another negative emotion (disgust), our work focused on studying the influence of neuroticism on verbal and physiological responses using specific and distinct categories of emotion (fear, disgust, and sadness).

To do so, we evaluated ANS responses by measuring the SCR, which mainly dependent upon arousal level [22]. We also assessed emotional expression by measuring the facial muscle activity of the corrugator (frowning), because its sensitivity to negative emotions allows the differentiation of emotions by valence.

Based on the literature and on the trait characteristics of neurotic subjects aforementioned [7], [17], [20], [21] we hypothesized that neurotics would have subjective responses, SCR, and corrugator activity that are more intense for fear and sadness as compared to low neurotics and to the other negative emotion.

## Methods

### Participants

Two hundred subjects were recruited via screening lists at the clinical investigation unit at the Timone Hospital in Marseille France and completed the NEO PI-R scale [22] to assess personality traits. The selection criterion for inclusion in the study was to obtain low neurotic scores (scores below 80) or high neurotic scores (scores higher than 110) defined by the rating scale of the NEO PI-R.

Among the subjects recruited, 33 (29 women and 4 men with a mean age of 27.5±10.7 years and a mean education of 10.3±2.7 years after grade 7) were included. Among them 15 were low neurotics (71.1±8.2) and 18 were high neurotics (125.4±9.6).

Participants also filled out the Beck Depression inventory (BDI) [23] and the State-Trait Anxiety Inventory (STAI-YB) [24] to assess their depression and anxiety levels. The investigation was carried out in accordance with the latest version of the Declaration of Helsinki. Participants provided written informed consent in accordance with the guidelines of the local ethics committee, CPP South Mediterranean 2. The ethics committee specifically approved this study.

### Stimuli

In the present study, 33 participants viewed a series of ten 45-seconds color films. The clips were chosen in a validation study to elicit three emotions (sadness, fear, or disgust). The excerpts had been previously validated with 15 healthy controls. Based on literature data distinghishing emotions according to their arousal level, and considering emotions of fear and disgust as more arousing than emotion of sadness [25]–[27], we selected six films that best fit our inclusion criteria (two for each emotional category): 1/ identification percentage higher than 80%; 2/ intensity of the induced emotion higher than 7; 3/ arousal level higher than 7 on the arousal scale for stimulating emotions (fear and disgust) and lower than 6 for non-stimulating ones (sadness). We thus selected excerpts that intensely induce the studied emotions and that are well differentiated on the arousal scale (Table 1).

**Table 1:** Results of the validation study.

Films	Identification of emotion (%)	Intensity of induced emotion	Arousal level
Famine in Biafra (sadness)	85±1.1	7.5±1.3	5.2±0.5
Stepmom (sadness)	100	7.3±1.2	5.5±0.8
A Tale of Two Sisters (fear)	100	7.2±0.5	7.2±1.5
A perfect murder (fear)	100	7.3±0.7	7.3±1.4
Accro (disgust)	100	8.4±0.6	7.2±1.4
surgery of the face (disgust)	100	7.2±1.4	7.3±0.8

Results of emotional identification, emotional intensity, and arousal level of the validation study. Means ± standard errors are represented.

The films were a report on the famine in Biafra by the Audiovisual National Institute, which shows conditions of extreme poverty and deprivation of Africans in Biafra, and an excerpt of the movie “Stepmom” by Columbus. The scene chosen from this film shows a mother with cancer announcing her upcoming death to her child. For fear, we used an excerpt from “A Tale of Two Sisters” by Jee-Woon, which begins with suspense and ends with an intense burst. The other film was an excerpt from “A perfect murder” by Davis. This film shows the sudden intrusion of an aggressor in the apartment of a young woman and then a violent aggression). For disgust, we used an excerpt from “Accro” by Mettling, a short film depicting a cannibalism scene, and an excerpt from a real-time surgery of the face performed in the Timone hospital in Marseille.

Participants were asked to watch the 45-second movies and to be aware of the resulting emotional experience to the best of their ability. The evaluation was explained at the beginning of the experiment and participants had to first identify the emotion by choosing between sadness, fear, disgust, or other emotion, and then identify its intensity and arousal respectively on a Likert-like scale from 0 to 10. Verbal evaluations were done at the end of each short movie presentation.

### Task procedure

Films were shown individually. They were fed into E-studio 2.2 software (E-Prime 2.2) and displayed on a 17-inch computer screen with a refresh rate of 100 Hz and 40W Yamaha NS10M Studio sound blasts, linked to a P2040 amplifier, at a sufficiently elevated and comfortable volume.

Participants were comfortably seated 50 cm from the screen. They were informed that the experiment was designed to study emotions using short films. Physiological sensors were attached to capture physiological activity. To assess subjective feelings, participants completed an arousal-rating scale (relaxing or arousing aspect of emotion) on a Likert-like scale from 0 to 10, during a post-film break. After this verbal evaluation, and after physiological parameters returned to baseline levels, the following film was displayed.

### Measures

Physiological data were acquired by two PCs, one running E-Prime (Psychology Software Tools, Inc., Pittsburgh, PA,) and the other Acqknowlege software (Biopac Systems, Inc., Goleta, CA). Physiological channels and rating dial information were recorded at a rate of 1000 Hz in continuous mode using the Biopac MP150 system.

SCR was measured in microsiemens using 5-mm inner diameter Ag/AgCl electrodes filled with isotonic electrode paste. Electrodes were attached to the volar surface of the second phalanx of the second and third right fingers in accordance with published guidelines [28].

Electromyogram (EMG) activity of corrugators muscle was also recorded. Facial frowning behavior was measured in microvolts by monitoring the activity of Corrugator Supercilii muscle on the left side of the face using surface Ag/AgCl electrodes (4 mm diameter; 10 mm distance between the two electrode centers) filled with conductive paste [29]. Electrodes were placed above the left eyebrow for assessment of Corrugator Supercilii muscle activity [29] (Fridlund & Cacioppo (1986). A ground electrode was placed on the lobe of the left ear.

### Statistical analysis of self-report and physiological data

Data of physiological parameters and verbal scoring were averaged for the two films per emotion. Data for EMG activity were calculated by subtracting the basal level from the mean level of the measurements during each of the 45-sec films. Level was considered basal for the 15 seconds prior to film onset when subjects were told to relax, and when physiological parameters were at baseline levels.

Data for SCR were obtained by averaging peak amplitudes of SCR during the 45-sec film excerpts. SCR below 0.01 µS were not considered. Artifact correction for SCRs consisted of a visual inspection of respiration and the manual exclusion of SCR that appeared to be influenced by coughs, sighs, or deep breath.

Statistical analyses were conducted using the 11.5.1 version of SPSS. A two-way repeated measures ANOVA was used with Emotion (sadness, fear, and disgust) as a within factor, and Personality (low neurotics subjects and high) as a between factor. Significant main effects were followed by post hoc tests using Bonferroni correction. A significance level of 0.05 was adopted in all tests.

## Results

### Psychometric Data

High neurotic subjects scored higher than low neurotic subjects on BDI (F (1,31) = 18.3; p<0.001), and STAI scales (F (1,31) = 71.9; p<0.001). Regarding the cut-off of pathology for each questionnaire, high neurotics were in the range of mild depression (6.7±1.2, mean±SD) on BDI, while they scored in the range of severe anxiety (51.4±1.2, mean±SD) on STAI scale.

### Arousal

**Figure 1:**
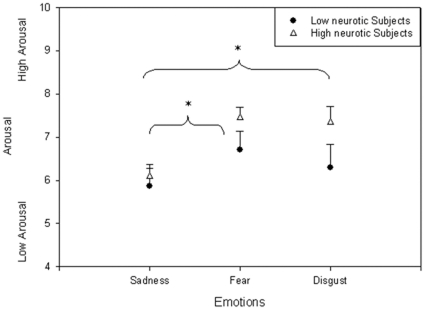
Arousal level. Plot of the intensity of Arousal level of the two populations as a function of the various emotions (mean and Standard Error bars). *p<0.05.

There was a significant main effect of Emotion on the arousal evaluation (F (2.62) = 6.3; p<0.01). Post hoc analysis revealed that fear and disgust excerpts were found more stimulating than sad extracts whatever the group, as shown in Figure 1.

### EMG corrugator muscle

**Figure 2:**
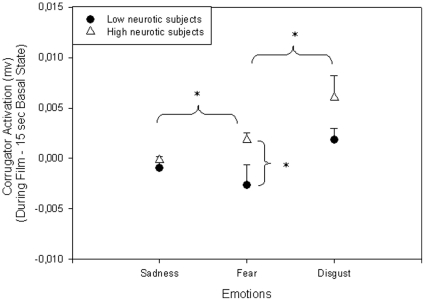
EMG corrugator muscle. Plot of the activity of the left corrugator frowning muscle of the two populations as a function of the various emotions (mean and Standard Error bars). *p<0.05.

There was a significant Population X Emotion interaction on the corrugator activity (F (2,62) = 5.2; p<0.05). When the emotion of fear was induced, the corrugator muscle activity was larger for high neurotic subjects than for low neurotic subjects (p<0.05) (Fig. 2).

High neurotic subjects (F (2,34) = 6.1; p<0.005) also had a larger corrugator muscle activity for emotions of disgust than emotions of fear and sadness and a greater activity for emotion of fear (p<0.05) than emotion of sadness.

**Figure 3:**
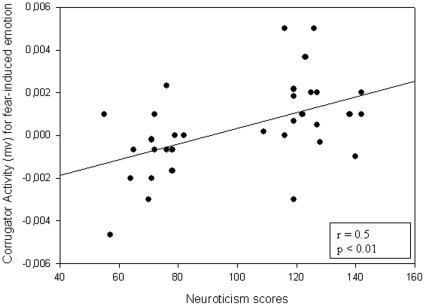
Correlation between corrugator activity for fear and neuroticism scores. Plot of the correlation indices between increasing left corrugator frowning muscle activity for fear-induced emotion and higher neuroticism scores on NEO PI-R.

There was a positive correlation between corrugator activity for fear and neuroticism scores on NEO PI-R (r = 0.5; p<0.01). This result indicated that the higher the neuroticism scores, the larger the corrugator activity for fear-induced emotion (Fig. 3).

### Electrodermal Conductance

**Figure 4:**
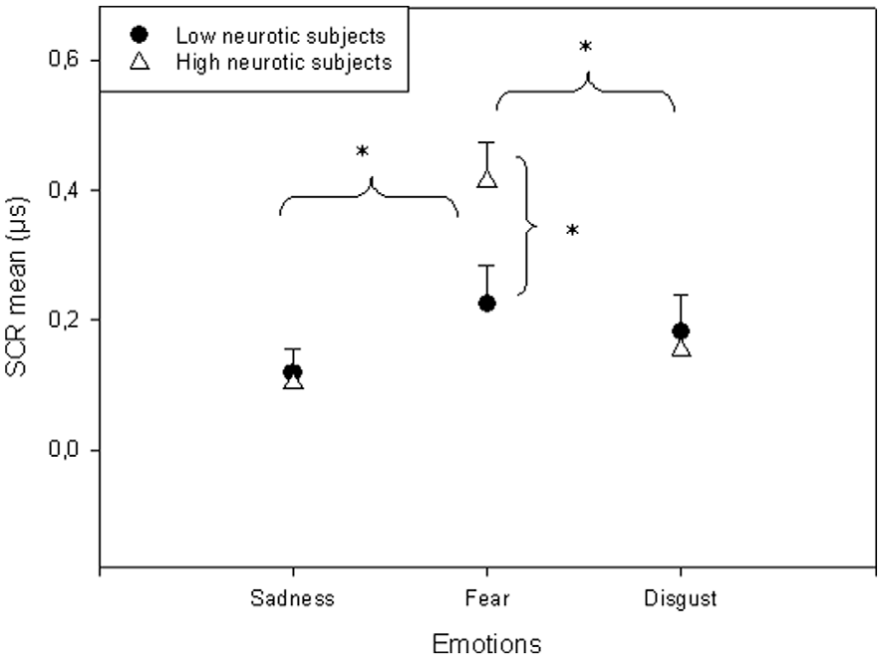
Electrodermal conductance. Plot of the mean amplitude of the electrodermal response of the two populations as a function of the various emotions (mean and Standard Error bars). *p<0.05.

There was a significant Population X Emotion interaction (F (2,62) = 9.7; p<0.0001) on electrodermal activity. When emotion of fear was induced, SCR was larger for high neurotic subjects than for low neurotic subjects (p<0.01) (Fig. 4).

In addition, the electrodermal response for high neurotic subjects (F (2,34) = 28.5; p<0.0001) was larger for emotions of fear as compared to emotions of sadness and disgust (p<0.0001).

**Figure 5:**
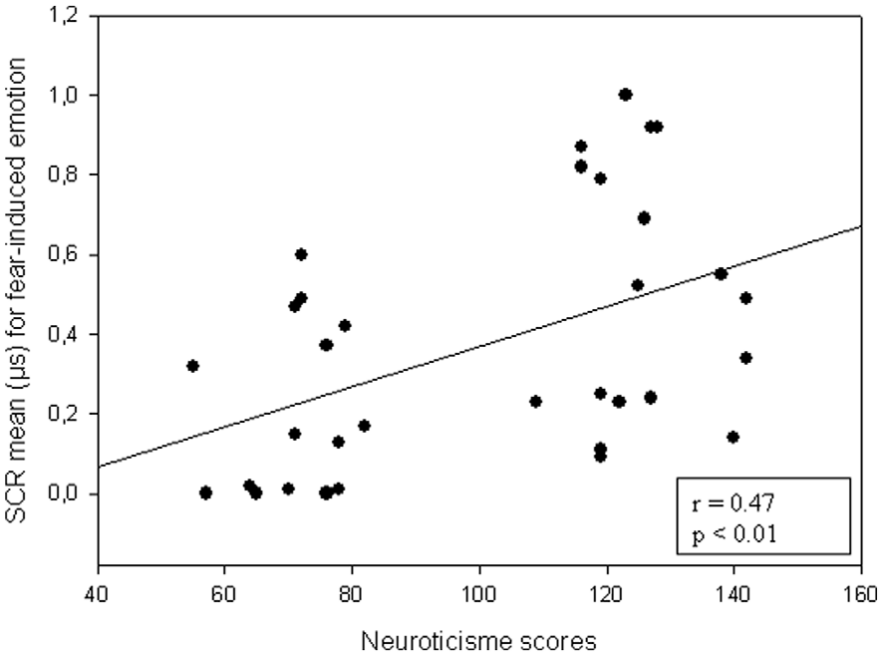
Correlation between mean SCR for fear and neuroticism scores. Plot of the correlation indices between increasing mean SCR for fear-induced emotion and higher neuroticism scores on NEO PI-R.

There was a positive correlation between SCR for fear and neuroticism scores on NEO PI-R (r = 0.47; p<0.01). This result indicated that the higher the neuroticism scores, the larger the SCR for fear-induced emotion (Fig. 5).

## Discussion

Our aim was to study the differences between high neurotic subjects and low neurotic subjects on verbal and physiological responses during the presentation of films inducing specific negative emotions of fear, sadness, and disgust.

Considering the emotional assessment, we first observed the effects of emotion induction on verbal measurement, with a higher arousal level for emotions of fear and disgust than for emotions of sadness in both groups. The two populations did not differ in verbal evaluation, but they were distinct in terms of physiological responses. The effect of neuroticism appeared stronger on physiological rating than on verbal rating, suggesting the existence of a dissocation between the way people rate their subjective experience and their inner physiological responses.

Physiological assessments showed greater SCR and corrugator muscle activity for high neurotics than for low neurotics when fear was induced but not sadness or disgust. This observation is supported by results of correlations suggesting that the higher the neuroticism scores, the greater the SCR and corrugator activity for fear-induced emotion. These results are in line with other findings that neurotic individuals exhibit both greater SCR and more sustained responses to emotionally unpleasant stimuli than do emotionally stable individuals [14]. Many studies have shown a clear association between neuroticism and measures of negative affect [6], [15], whereby neurotics easily experience feelings such as anxiety, stress, depression, and fear [30]. Our study implies that the difference between high and low neurotics may lie in fear. This novel result corroborates the postulate that neuroticism predicts higher threat appraisals [17]. Those subjects high in neuroticism were more susceptible to negative affect, particularly when they construed an impending stressor as threatening. It has been postulated that in neuroticism, the tendency to experience distress and negative affect [15] constitutes a psychological readiness to perceive threat [16].

At the cerebral level, functional magnetic resonance imaging (fMRI) studies have provided evidence that the experience of fear relies upon activity of the amygdala, the anterior cingular cortex, and the medial prefrontal cortex [31]–[33], which are also associated with neuroticism [34]–[37]. A recent study measuring functional connectivity has shown that high neurotic participants display diminished anterior cingular cortex control over the amygdala when processing fearful faces [9]. These neuroimaging studies converge in suggesting that the mechanisms involved in the overall response to fear are altered in neuroticism. These specific structures modulate the autonomic nervous system and influence measures of the peripheral nervous system [38], [39]. This interaction between the central nervous system and autonomic nervous system may therefore explain the increase in SCR and corrugator activity for neurotic subjects when fear emotion is induced.

The literature has established a link between neuroticism and depression [18], [19] that we expected would influence the physiological responses to sadness. Yet we did not find any differences between low and high neurotic subjects in sadness as previously hypothetized. This lack of difference may be explained by the fact that neurotics had only a mild level of depression on Beck scale, whereas they had a high anxiety level. Anxiety is linked to disturbances in fear responses [31], [40], which could account for our results of an elevation of SCR and Corrugator activity for fear. Both physiological measures, SCR and corrugator activity, that we used in this study allowed us to respectively distinguish emotions based on their arousal and valence levels. The SCR is described as being sensitive to the arousal level of emotions, but we did not find correlations between the arousal level of emotions and SCR in our study. That we did not find any differences in SCR for sadness could not be completely explained by the low level of arousal this emotion brings. Indeed, considering SCR for each emotion, all three emotions produced significant electrodermal responses (i.e. SCR higher than 0.01 µs as presented in the Methods section), indicating that SCR is reactive to all emotions. In this regard, we could have found differences between high and low neurotics for sadness or disgust. Yet, the difference is only related to fear.

Another major point is the increased corrugator activity for the emotion of disgust as compared to emotions of fear and sadness, and the presence of an increased SCR for the emotion of fear as compared to emotions of sadness and disgust for high neurotic subjects. This differentiation between emotions was not found for low neurotic subjects. These results are in accordance with physiological studies in healthy subjects, showing that the emotion of disgust generated the most significant activation of facial corrugator muscle as compared to other negative emotions [41], [42]. Moreover, William et al. (2001) [43] observed a correlation between the amplitude of the SCR and the arousal level of stimuli presented. SCR increased for highly stimulating extracts, especially for fear-induced emotion [23], [44]. According to our results, it is striking that somatic markers seem to be less sensitive for low neurotic subjects to distinguish emotions and/or that those markers are more sensitive in high neurotic individuals.

### Conclusion

Added to decades of research evidencing that individuals higher in neuroticism experience more intense emotional reactions to even minor stressors than other individuals [6], our results reveal that high neurotic individuals show specifically greater expressive and SCR reactivity to fearful stimuli.

The present study shows that neuroticism can modulate emotional reactivity, especially when fear rather than sadness or disgust is generated. This result is in accordance with fMRI studies on neuroticism. The modulation of autonomic activity in response to threat/fear by neurotics may be part of what leads to increased vulnerability to anxious psychopathologies such as PTSD (post traumatic stress disorder), which is characterized by high level of neuroticism [45] and disturbances of the mechanisms involved in the overall response to fear [46]. Further work should be conducted to investigate physiological and cerebral responses involved in fear in neurotics without PTSD vs. those with PTSD in order to observe whether the same mechanisms are involved.
